# Post-translational Modifications in Oral Bacteria and Their Functional Impact

**DOI:** 10.3389/fmicb.2021.784923

**Published:** 2021-12-02

**Authors:** Qizhao Ma, Qiong Zhang, Yang Chen, Shuxing Yu, Jun Huang, Yaqi Liu, Tao Gong, Yuqing Li, Jing Zou

**Affiliations:** ^1^State Key Laboratory of Oral Diseases, National Clinical Research Center for Oral Diseases, West China Hospital of Stomatology, Sichuan University, Chengdu, China; ^2^Department of Pediatric Dentistry, West China Hospital of Stomatology, Sichuan University, Chengdu, China

**Keywords:** post-translational modifications, bacteria, physiology, bacterial virulence, *Streptococcus mutans*, *Porphyromonas gingivalis*

## Abstract

Oral bacteria colonize the oral cavity, surrounding complex and variable environments. Post-translational modifications (PTMs) are an efficient biochemical mechanism across all domains of life. Oral bacteria could depend on PTMs to quickly regulate their metabolic processes in the face of external stimuli. In recent years, thanks to advances in enrichment strategies, the number and variety of PTMs that have been identified and characterized in oral bacteria have increased. PTMs, covalently modified by diverse enzymes, occur in amino acid residues of the target substrate, altering the functions of proteins involved in different biological processes. For example, Ptk1 reciprocally phosphorylates Php1 on tyrosine residues 159 and 161, required for *Porphyromonas gingivalis* EPS production and community development with the antecedent oral biofilm constituent *Streptococcus gordonii*, and in turn Php1 dephosphorylates Ptk1 and rapidly causes the conversion of Ptk1 to a state of low tyrosine phosphorylation. Protein acetylation is also widespread in oral bacteria. In the acetylome of *Streptococcus mutans*, 973 acetylation sites were identified in 445 proteins, accounting for 22.7% of overall proteins involving virulence factors and pathogenic processes. Other PTMs in oral bacteria include serine or threonine glycosylation in Cnm involving intracerebral hemorrhage, arginine citrullination in peptidylarginine deiminases (PADs), leading to inflammation, lysine succinylation in *P. gingivalis* virulence factors (gingipains, fimbriae, RagB, and PorR), and cysteine glutathionylation in thioredoxin-like protein (Tlp) in response to oxidative stress in *S. mutans*. Here we review oral bacterial PTMs, focusing on acetylation, phosphorylation, glycosylation, citrullination, succinylation, and glutathionylation, and corresponding modifying enzymes. We describe different PTMs in association with some examples, discussing their potential role and function in oral bacteria physiological processes and regulatory networks. Identification and characterization of PTMs not only contribute to understanding their role in oral bacterial virulence, adaption, and resistance but will open new avenues to treat oral infectious diseases.

## Introduction

Post-translational modifications (PTMs) expand nature’s inventory of the 20 proteinogenic amino acids by inducing covalent modifications in amino acid residues (Beck-Sickinger and Mörl, 2006). These modifications alter protein functions, such as enzymatic activity, localization, stability, or interactions with other molecules across all domains of life. As the main component of oral microflora, oral bacteria depend on PTMs to quickly respond to external stimuli and regulate their physiological processes. In oral bacterial species, common modifications include phosphorylation, acetylation, glycosylation, citrullination, succinylation, and glutathionylation (Wu et al., 2019; Zeng et al., 2020).

Post-translational modifications modulate bacterial physiological processes and regulate bacterial adaptability to the environment. The complex and harsh oral environment connects the internal body with the external environment. Due to the particularity of the oral anatomy, the oral bacteria are susceptible to host conditions, e.g., dietary habits, immune responses, pH, poor oral hygiene, oxygen tension, and lifestyle. When oral bacteria encounter rapidly changing and hostile environmental stresses, PTMs are a highly efficient mechanism to regulate protein function (e.g., from inactive to active) compared with the energy-extensive processes of gene expression, protein synthesis, and protein degradation. For example, sensor histidine kinase is autophosphorylated in two-component regulatory systems (TCS), quickly activating the downstream response regulator (RR). Such “fast-switching” events allow oral bacteria to quickly respond to external stimuli, which is more efficient than the regulation of gene expression. Under oxidative stress, proteins could use *S*-glutathionylation to maintain intracellular redox homeostasis (Loi et al., 2015).

In addition, PTMs play an essential role in regulating bacterial virulence, subsequently affecting oral homeostasis and disease conditions. For example, oral bacteria facilitate bacterial colonization and the accumulation of multicellular clusters by glucosyltransferases to synthesize extracellular polysaccharides (EPS), the major constituents of the plaque biofilm. Notably, the acetylation level of glucosyltransferases is closely associated with their activity. In addition to local influences, pathogenic bacteria in the oral cavity can gain access to the bloodstream, causing systemic infections by secreting virulence factors (Liu et al., 2012; Blod et al., 2018; Lira-Junior and Boström, 2018; Philip et al., 2018; Reddy et al., 2018; Saikaly et al., 2018). Multiple PTMs occur in these secreted virulence factors. For example, the peptidylarginine deiminase (PAD) secreted by *Porphyromonas gingivalis* not only could citrullinate arginine-specific protease A (RgpA) and arginine-specific protease B (RgpB), the important virulence of periodontitis but citrullinate human host proteins that are closely related to systemic diseases. The glycosylation of serine-rich repeat (SRR) glycoprotein gordonii surface protein (GspB) in *Streptococcus gordonii* contributes to the infective endocarditis (IE) by binding to human platelets (Seepersaud et al., 2020).

Unlike mRNA or protein synthesis, PTMs are not templated but usually rely on recognition and modification by the relevant enzyme. Most PTMs are reversible and dynamic, i.e., modified chemical groups can be placed onto or removed from the polypeptide chain by specialized enzymes, such as the kinases Stk1 and phosphatases Stp1 in *Streptococcus agalactiae* and acetyltransferases Pat and deacetylase CobB in *P gingivalis*. In addition to the specialized enzymes, some chemical compounds are also involved in protein modifications. For example, acetyl-coenzyme A (Ac-CoA) directly increased the acetylation of RprY in *P. gingivalis*. Succinylation was also mediated non-enzymatically with succinyl-CoA as the donor (Wagner and Payne, 2013; Wagner and Hirschey, 2014).

Moreover, proteins commonly carry multiple modifications, and some residues can simultaneously carry several modifications. For instance, acetylation and succinylation in *P. gingivalis* are extensively overlapped. RprY could be both acetylated and phosphorylated. Such PTM crosstalk can mediate complementary or opposing effects, resulting in a complex combination of the structure and function of the target protein, which illustrates the complexity and flexibility of the underlying regulatory processes.

With the development of novel enrichment strategies of PTM sites in past decades, new PTMs are constantly being discovered, with new targets and sites of known PTM in oral bacteria. Consequently, research into PTMs is burgeoning and growing, particularly in regulating bacterial physiological processes (Walsh et al., 2005; Cain et al., 2014). This review introduces the significant types of PTMs in oral bacteria and discusses their roles in bacterial physiological and pathological processes (Table 1).

**TABLE 1:** Post-translational modifications (PTMs) and their functional roles in oral bacteria.

**PTM**	**Protein**	**Target residue**	**Function**	**Organism**	**References**
**Protein phosphorylation**					
Histidine phosphorylation	VicK	His residue	VicR, regulating the expression of *gtfB/C*	*S. mutans*	Wang et al., 2021
	ComD	His residue	ComE, inducing bacterial competence and exogenous DNA uptake	*S. mutans*	Shepherd et al., 2012
	NsrS	His residue	NsrR, regulating the expression of *nsrX*	*S. mutans*	Kawada-Matsuo et al., 2013
	LcrS	His residue	LcrR, regulating the expression of *lctFEG*	*S. mutans*	Kawada-Matsuo et al., 2013
	HaeS	His226	HaeR, regulating iron uptake/acquisition genes	*P. gingivalis*	Huynh et al., 2010; Willett and Kirby, 2012
	PorY	His residue	PorX, type IX secretion system	*P. gingivalis*	Kadowaki et al., 2016
	FimS	His residue	FimR, regulating the expression of *fimA*	*P. gingivalis*	Nishikawa et al., 2004
Serine/threonine phosphorylation	CovR	Thr65	Regulate expression of CAMP factor	*S. agalactiae*	Lin et al., 2009
	PknB	Ser and/or Thr residue	Regulating the activity of PppL, VicKR, and ComDE	*S. mutans*	Banu et al., 2010; Zhu and Kreth, 2010
	SpxB	Thr409, 415, 508	Mediating pyruvate conversion	*S. sanguinis*	Mu et al., 2021
	PGN_0375, PGN_0500, PGN_0724, PGN_0733, PGN_0880	Ser and/or Thr	Not determined	*P. gingivalis*	Izumigawa et al., 2016
Tyrosine phosphorylation	Php1	Tyr159, 166	Exopolysaccharide production, proteinases phosphorylation	*P. gingivalis*	Jung et al., 2019
	RprY	Tyr41	Type IX secretion system	*P. gingivalis*	Shen et al., 2020
	SpxB	Tyr409, 415, 588	Intraspecies and interspecies competition	*S. sanguinis*	Mu et al., 2021
**Protein acetylation**	RgpA, RgpB, Kgp	Lysine residue	Activation/maturation of gingipains	*P. gingivalis*	Mishra et al., 2018
	RprY	Lysine residue	Bacterial survival	*P. gingivalis*	Li et al., 2018
	GtfB, GtfC, GtfD,	Lysine residue	Biofilm formation	*S. mutans*	Lei et al., 2020
**Protein glycosylation**					
*N*-glycosylation	GspA	Asn residue	Bacterial survival	*A. oris*	Li et al., 2004; Dige et al., 2009
	CpsA	Asn residue	CPS synthesis	*S. agalactiae*	Stefanovic et al., 2021
*O*-glycosylation	LPS	Ser and/or Thr residue	Collagen-binding activity	*A. actinomycetemcomitans*	Schaffer and Messner, 2017
	Cnm, WapA	Ser and/or Thr residue	Adhesion	*S. mutans*	Aviles-Reyes et al., 2018
**Protein citrullination**	PPAD	Arg residue	Bacterial survival	*P. gingivalis*	Stobernack et al., 2016
	Rgp	Arg residue	Inflammatory responses	*P. gingivalis*	Konig et al., 2015
**Protein succinylation**	Fimbriae, RagB, PorR	Lysine residue	Inflammatory responses	*P. gingivalis*	Wu et al., 2019
**Protein *S*-glutathionylation**	Tlp	Cys41	Maintaining intracellular redox homeostasis	*S. mutans*	Li et al., 2020

## Major PTMs in Oral Bacteria and Their Functional Impact

### Protein Phosphorylation

**FIGURE 1:**
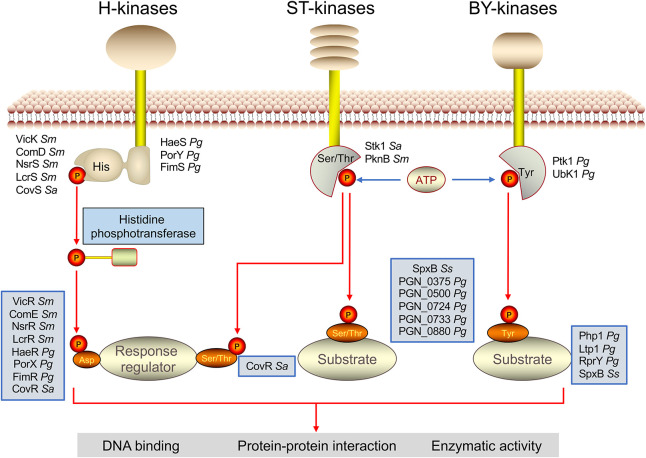
Kinase-mediated protein phosphorylation in oral bacteria. Protein phosphorylation is involved in the kinase-catalyzed addition of phosphate to amino acid side chains of histidine (His), serine (Ser), threonine (Thr), and tyrosine (Tyr). The histidine phosphorylation in oral bacteria belongs to the two-component systems (TCS) composed of a membrane-located sensor His kinase and a cytoplasmic response regulator. The former comprises an extracellular sensory domain linked to a cytoplasmic domain required for catalysis and dimerization. The phosphate group is subsequently transferred from histidine to an aspartate residue of the latter. The activated response regulator mediates the physiological processes, mostly by regulating the transcription of some target genes. Bacterial Hanks-type ST-kinases are most commonly transmembrane proteins containing an extracellular domain binding to ligands and a cytosolic protein kinase domain. The kinase domain phosphorylates the serine and threonine residues of target substrates using the phosphate group of ATP as the donor. The protein tyrosine phosphorylation is catalyzed by the BY kinase family, which has thus far been found only in bacteria. BY kinases possess a transmembrane domain that functions not only as a kinase anchor but also as a sensor domain and an intracellular catalytic domain that can phosphorylate target proteins on tyrosine using the phosphate group as the donor. *Pg* for *P. gingivalis*, *Sm* for *S. mutans*, *Sa* for *S. agalactiae*, and *Ss* for *S. sanguinis*.

Common protein phosphorylation involves kinase-catalyzed addition of phosphate to amino acid side chains of histidine (His), aspartate (Asp), serine (Ser), threonine (Thr), and tyrosine (Tyr) in oral bacteria. Protein phosphorylation can be readily reversed by phosphatase-mediated hydrolysis (dephosphorylation) to regenerate unmodified hydroxyls, i.e., changing the residue from hydrophobic to hydrophilic (Mijakovic et al., 2016). Therefore, reversible protein phosphorylation can regulate the characteristics of proteins rapidly and react quickly to external stimuli. There are many donor sources of phosphate groups, the most utilized of which are active phosphate groups and adenosine triphosphate (ATP). Most phosphorylated side chains or kinases are residue-specific, and different phosphorylated amino acid side chains have different chemical stability and distinct impacts on hemostasis and quality control. Protein phosphorylation is widely involved in metabolic processes, including cell survival, virulence factor, and environmental adaptation in oral bacteria. Different phosphorylation modifications will be reviewed using the families of kinases and the residues they phosphorylate in this part (Figure 1).

#### Histidine Phosphorylation

The best-characterized histidine phosphorylation in oral bacteria belongs to the so-called two-component systems (TCSs), consisting of a membrane-located sensor His kinase (HK) and a cytoplasmic RR. The former is autophosphorylated on the His residue, and the phosphoryl group is subsequently transferred to aspartate (Asp) residue of the response regulator receiver domain, or additional domains, such as DNA-binding or other effector domains, to translate the sensed signal in response to environmental stimuli.

Two-component systems identified in oral bacteria play a crucial role in responding to environmental stresses and regulating bacterial virulence factors. VicRK, a member of TCS in *Streptococcus mutans*, is associated with biofilm formation. VicK can regulate the phosphorylation of the regulator VicR upon signal-induced autophosphorylation. Wang et al. (2021) reported that proline at position 222 in the PAS domain of VicK is responsible for its phosphatase activity, and the VicK^P222A^ mutation inhibited its phosphatase activity and subsequently attenuated the phosphorylation of VicR that regulated the expression of *gtfB/C*, reducing the biofilm formation and bacterial growth in *S. mutans*. In addition, competence stimulating peptides (CSPs) could activate the His kinase ComD, which phosphorylates the response regulator ComE, leading to gene expression and induction of competence. Thus, competent *S. mutans* can take up exogenous DNA to develop bacterial resistance to antibiotics (Shepherd et al., 2012). Additional TCSs identified in *S. mutans* include NsrRS and LcrRS. NsrRS and LcrRS individually regulate the expression of *nsrX* and the ABC transporter *lctFEG*, which are associated with resistance against the lantibiotics nisin A and nukacin ISK-1, respectively (Kawada-Matsuo et al., 2013). In *P. gingivalis*, TCS HaeSR regulates numerous genes of iron uptake/acquisition that encode transporters and metabolic functions, which is vital for its survival. It was further demonstrated that ATP serves as the phosphodonor to phosphorylate the HaeS at the conserved histidine 226 residue (Huynh et al., 2010; Willett and Kirby, 2012). The PorXY has been demonstrated as the TCS and regulates the type IX secretion system in *P. gingivalis* (Kadowaki et al., 2016). In addition, TCS FimSR regulates the expression of *fimA* encoding the fimbrilin protein subunit of fimbriae, which is associated with biofilm formation and binding to other bacteria (Nishikawa et al., 2004).

#### Serine/Threonine Phosphorylation

The signaling systems composed of Hanks-type serine/threonine kinases (STKs) and phosphatases (STPs) also play an essential role in bacterial regulatory networks. STKs are either membrane or cytoplasmic proteins containing a catalytic domain involved in the phosphorylation of Ser/Thr residue and additional subdomains responsible for regulating their activities, binding to ligands, and protein-protein interactions. Similarly, STKs and STPs are engaged in responses to changing environmental conditions.

Although the systems are not involved in His phosphorylation, they can affect gene expression by directly phosphorylating TCS response regulators. It is reported that the STK Stk1 from *S. agalactiae* can phosphorylate the CovR response regulator at the Thr65 and then downregulate transcription of CAMP (Christie, Atkins Munch-Peterson) factor, a cytotoxin (Rajagopal et al., 2006; Lin et al., 2009). Differently, as a member of TCSs, the phosphorylation of CovR can be activated by HK CovS in *S. agalactiae* (Lamy et al., 2004; Jiang et al., 2005). It is reported that CovS activates the response regulator CovR by transphosphorylation at the aspartate residue 53 (D53) and subsequently the phosphorylated CovR upregulates the expression of CAMP (Rajagopal et al., 2006). In this case, CovR can be phosphorylated by HK CovS at D53 and also can be phosphorylated by STK Stk1 at Thr65. While the phosphorylation of CovR at Thr65 leads to inhibition of expression of CAMP, phosphorylation of CovR at D53 leads to activation. Apart from STK, cognate STPs Stp1 are engaged in this process. In addition, *S. mutans* contains one STK and one STP, referred to as PknB and PppL, respectively. It has been reported that PknB regulates biofilm formation, bacteriocin production, and cell wall metabolism by modulating the activity of the TCS VicKR and ComDE in *S. mutans* (Banu et al., 2010; Zhu and Kreth, 2010). The Ser/Thr phosphorylation is engaged in other metabolic processes in oral bacteria in addition to regulating TCS. Streptococcal pyruvate oxidase (SpxB), a hydrogen peroxide-generating enzyme, was confirmed to have Thr phosphorylation. The mutated SpxB protein, including the substitution of Thr at positions 409, 415, and 508 with Asp, exhibited decreased solubility *in vivo*, contributing to bacterial growth, colony morphology, and hydrogen peroxide (H_2_O_2_) production by mediating pyruvate conversion in *Streptococcus sanguinis* (Mu et al., 2021). Five phosphoproteins with Ser and/or Thr were identified in *P. gingivalis*, referred to as PGN_0375 (phosphoribulose/uridine kinase), PGN_0500 (methylmalonyl-CoA decarboxylase), PGN_0724 (NAD-dependent hydroxybutyrate dehydrogenase), PGN_0733 (alpha-glucan phosphorylase), and PGN_0880 (tryptophanase), using the phosphate-affinity chromatography and anti-Ser and -Thr antibodies. However, the specific functions and regulatory mechanisms should be further explored (Izumigawa et al., 2016).

#### Tyrosine Phosphorylation

Protein tyrosine phosphorylation is catalyzed by the tyrosine (BY) kinase family, which has thus far been identified only in bacteria (Mijakovic et al., 2016). A BY kinase possesses a transmembrane domain that can function not only as a kinase anchor but also as a sensor domain and an intracellular catalytic domain that binds ATP and transfers its phosphate to the hydroxyl unit of the tyrosine residue (Sun et al., 2020). The catalytic domain is defined by the canonical Walker A (P-loop) and B motif. In addition, some BY kinases also contain an additional Walker A′ motif. Walker A and B motifs are usually found in nucleotide-binding proteins; however, Walker A′ motif is found in certain kinase phosphorylation low-molecular-weight (LMW) substrates such as Ltp1 in *P. gingivalis* (Liu et al., 2017).

BY kinases have an essential role in the regulation of various signal transduction pathways via substrate phosphorylation. It has been reported that Ptk1, a BY kinase, can phosphorylate Php1 on Tyr residues 159 and 166, and Ltp1, which are responsible for exopolysaccharide production and regulating pathway constraining *P. gingivalis*–*S. gordonii* community development, and gingipain proteinases Kgp, RgpA, and RgpB phosphorylation, respectively (Jung et al., 2019). *In vivo*, loss of Php1 significantly reduces the ability of *P. gingivalis* to induce bone loss.

RprY, an orphan response regulator, could be phosphorylated on Tyr residue (Li et al., 2018). The mutated phenylalanine (F) at Tyr41 (Y41F) diminished its affinity for the promoter region of *mfa1*, responsible for the expression of genes encoding the type IX secretion system (T9SS) machinery and virulence factors secreted through the T9SS, including gingipain proteases and *P. gingivalis*-derived peptidylarginine deiminase (PPAD). Consistently, the RprY with a Y41F substitution impaired *P. gingivalis* virulence in a murine model of alveolar bone loss (Shen et al., 2020). Furthermore, a recent report showed that the Tyr phosphorylation of RprY is mediated by the Ubiquitous bacterial Kinase 1 (UbK1) (Perpich et al., 2021). In addition to Thr phosphorylation of SpxB, the Tyr phosphorylation at positions 409, 415, and 588 was also identified, with a possibly significant role in intraspecies and interspecies competition in *S. sanguinis* (Mu et al., 2021).

### Protein Acetylation

**FIGURE 2:**
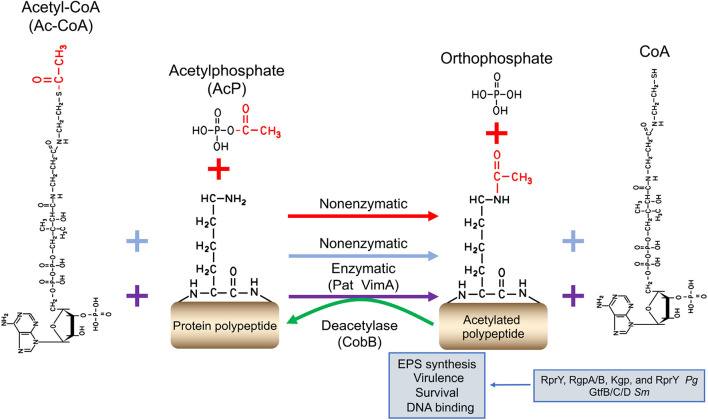
The regulatory mechanism of protein acetylation. Protein acetylation is finely regulated via two distinct mechanisms: enzymatic and non-enzymatic acetylation (chemical acetylation). Enzymatic acetylation relies on protein acetyltransferases to transfer the donation of the acetyl donor from Ac-CoA (purple plus and arrow signs). Non-enzymatic acetylation needs Ac-P (red plus and arrow signs) or Ac-CoA (blue plus and arrow signs) to serve as the acetyl donor. The reversibility of lysine acetylation is implemented by deacetylases. The acetyl functional group is shown in red. *Pg* for *P. gingivalis*, and *Sm* for *S. mutans*.

Protein acetylation is another major regulatory PTM found ubiquitously in oral bacteria. The protein acetylation modifications are finely regulated by two distinct mechanisms: enzymatic and non-enzymatic acetylation (chemical acetylation; Figure 2). Enzymatic acetylation relies on protein acetyltransferase to transfer the acetyl group from Ac-CoA to lysine residue (Starai and Escalante-Semerena, 2004). Non-enzymatic acetylation requires acetyl phosphate (AcP) or Ac-CoA to serve as the donor of the acetyl group. Lysine acetylation is carried out covalently by adding an acetyl group to a lysine residue, changing the biochemical characteristics of target protein, such as their charge, stability, and/or conformation, and subsequently, altering the protein biological activity (Carabetta and Cristea, 2017; Sang et al., 2017).

Lysine acetylation has been shown to play an essential role in bacterial survival and virulence. Butler et al. (2015) identified 130 lysine-acetylated peptides from 92 *P. gingivalis* proteins, including the three gingipains RgpA, RgpB, and Kgp in a *P. gingivalis* W40 acetylome study. In a previous report, the inactivation of *vimA*, a putative acetyltransferase gene, resulted in the late-onset gingipain activity, compared with the wild-type W83, which is further explained in the following report. PG1842 (a functional VimA homolog) and VimA can acetylate pro-RgpB at residues K247 and K248, contributing to the activation/maturation of gingipains in *P. gingivalis* (Mishra et al., 2018).

In the acetylome of *S. mutans*, 973 acetylation sites were identified in 445 proteins, accounting for 22.7% of overall proteins, among which 617 acetylation sites were quantified in 302 proteins. Notably, the acetylation levels of glucosyltransferase B (GtfB), glucosyltransferase C (GtfC), and glucosyltransferase D (GtfD) in the biofilms decreased by 29, 22, and 37%, respectively, compared to planktonic conditions (Lei et al., 2020). Gtfs are the critical virulence factors by which *S. mutans* can utilize sucrose to synthesize EPS, promoting biofilm formation, the initiating factor for caries. The results from the acetylome study indicate that the acetylation of Gtfs is an important mechanism of regulating their catalytic activity and subsequently the biofilm formation.

Lysine acetylation is reversed by deacetylases. Lysine deacetylases (NAD^+^-dependent sirtuins; for example, CobB) can enzymatically remove the acetyl group from lysine side chains. Reports on the characterization of acetyltransferases and deacetylases emerge continuously, such as the acetyltransferase Pat and the deacetylase CobB from *P. gingivalis*. Li et al. (2018) reported that RprY could be modified by Pat with Ac-CoA serving as the acetyl group donor, and the acetylated RprY could be reversely deacetylated by CobB. Subsequently, the acetylation of RprY diminished its ability to bind the promoter DNA of *nqrA* (PGN_0115: Na^+^-translocating NADH-quinone reductase subunit A), affecting bacterial survival in oxidative stress.

### Protein Glycosylation

**FIGURE 3:**
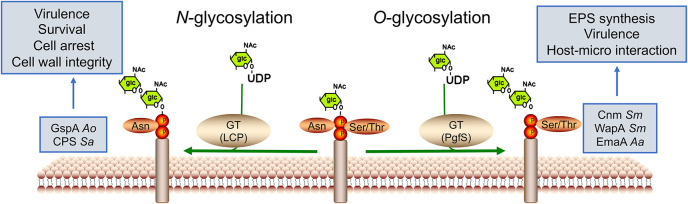
Glycosyltransferase-mediated protein glycosylation in oral bacteria. Protein glycosylation entails the covalent attachment of a glucan to target amino acid residues to form glycoproteins by glycosyltransferases (GTs). The donor is phosphate-activated sugar linked to nucleotides (UDP-). Glycosylation is commonly found on the bacterial surface. Based on the glycosidic linkage, protein glycosylation can be categorized into two major types: *N*-linkages and *O*-linkages, occurring in asparagine and serine or threonine residues, respectively. *Ao* for *A. oris*, *Sa* for *S. agalactiae*, and *Sm* for *S. mutans*.

Protein glycosylation is considered one of the most abundant forms of PTMs and identified as a critical regulator in bacterial physiology, such as communication, adhesion, and virulence (Nothaft and Szymanski, 2010). It entails the covalent attachment of a glycan group to the amino acid residue to form glycoproteins (Figure 3). The glycan donors are phosphate-activated sugars linked to nucleotides (UDP-, GDP-, and ADP-) or dolichol-related lipid groups. Protein glycosylation can be categorized into two major types, *N*-linkages and *O*-linkages, based on the glycosidic linkage; however, Cys-*S*-linked and Trp-*C*-linked glycoproteins are also known (Stepper et al., 2011; Yoshimoto et al., 2021). *N*-glycosylation binds glycans to the asparagine (Asn) residues, while *O*-glycosylation occurs at Ser or Thr residues. Protein glycosylation results from the modification of glycosidases and glycosyltransferases, whose activities depend on various bacterial processes and environmental stresses (Schaffer and Messner, 2017).

#### *N*-glycosylation

*N*-glycosylation generally contains a common pentasaccharide core with *N*-acetyl glucosamine (GlcNac) serving as the attachment site to the Asn residue in the specific protein. *N*-glycosylated proteins are commonly found on bacterial surfaces, as exemplified by the LyrT-A-Psr (LCP) transferases in *Actinomyces oris* (Gosschalk et al., 2020). *A. oris* (formerly known as *Actinomyces naeslundii*), a pioneer of the oral cavity, is found in significant numbers in dental plaque, as well as on mucosal surfaces in adults (Ellen, 1976; Li et al., 2004; Dige et al., 2009). LCP transferase can glycosylate GspA that regulates sortase enzyme (SrtA) activity, vital for bacterial survival, and can arrest *A. oris*. In *S. agalactiae*, CpsA is covalently attached to GlcNac of the peptidoglycan (PGN) backbone and is involved in cell wall maintenance and capsular polysaccharide (CPSCPS) synthesis (Stefanovic et al., 2021). Although known for many years, the study of *N*-glycosylation in oral bacteria is limited, necessitating further exploration.

#### *O*-glycosylation

*O*-glycosylation is usually connected to hydroxyl oxygen of Ser or Thr residues through *N*-acetylgalactosamine (GalNac). The main types of *O*-glycosylation are cell-surface proteins, such as adhesins. The extracellular matrix protein adhesin A (EmaA), an adhesin, has been reported to be linked to the *O*-polysaccharide of the bacterium’s LPS, maintaining collagen-binding activity in *Aggregatibacter actinomycetemcomitans* (Schaffer and Messner, 2017). The collagen- and laminin-binding protein (Cnm) and wall-associated protein A (WapA) could be glycosylated by glycosyltransferase PgfS, increasing their proteolytic stability and contributing to the adhesion of *S. mutans* to the host (Aviles-Reyes et al., 2018).

In addition to these *O*-GalNAc glycans, many proteins also have a single GlcNac covalently linked to Ser or Thr residues, similar to the glycosylation of SRR glycoproteins. SRR glycoproteins are a family of adhesins expressed by a wide range of oral bacteria, including *S. gordonii*, *Streptococcus parasanguinis*, *S. agalactiae*, and *Lactobacillus* species. The glycosylation of SRR glycoproteins contributes to colonization and virulence in various infections by binding to diverse host ligands (Chaze et al., 2014; Lee et al., 2014; Bleiziffer et al., 2017; Jiang et al., 2017). In *S. gordonii*, the SRR glycoprotein GspB can be glycosylated by glycosyltransferase GtfAB by transferring GlcNAc to Ser and Thr residues of the SRR domains, reducing its binding ability to human platelets. Moreover, GspB can be glycosylated by two additional Gtfs (Nss and Gly). After transferring Glc to GlcNAc by Nss, Gly transfers the second Glc residue, subsequently generating a trisaccharide (Seepersaud et al., 2020).

### Protein Citrullination

**FIGURE 4:**
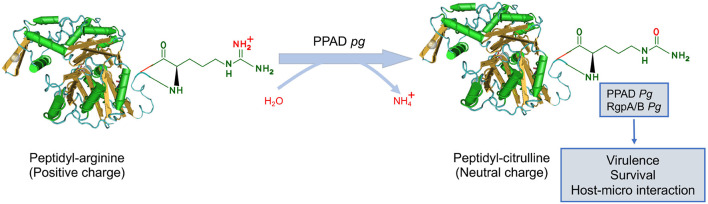
The regulatory mechanism of protein citrullination. Protein citrullination is catalyzed by peptidylarginine deiminases (PADs), resulting in the conversion of arginine on the functional peptide to citrulline (defective protein), which leads to the loss of positive charge. At present, *P. gingivalis* is the only known pathogen that produces PAD, referred to as PPAD. PPAD not only citrullinates proteins in bacteria but also specific human host proteins by gaining access to the bloodstream. *Pg* for *P. gingivalis*.

Citrullination is an essential PTMs in which peptidylarginine is catalyzed to peptidyl-citrulline by PADs. The reaction is referred to as delamination due to the exchange of an imine for a carbonyl group (Tilvawala and Thompson, 2019). The PAD-catalyzed reaction involves the hydrolysis of the guanidinium group of an arginine residue, leading to the loss of positive charge, generation of two hydrogen bond donors, and urea (Figure 4). Eventually, citrullination alters the charges of the substrate protein, possibly leading to significant changes in its structure and function (Wegner et al., 2010a). Although the existence of “decitrullinase” has been hypothesized, no such enzyme has been identified. The dysregulated PAD activity is observed in numerous inflammatory diseases including rheumatoid arthritis (RA), Alzheimer’s disease, atherosclerosis, and periodontal diseases (PDs) (Vossenaar et al., 2003; Ishigami et al., 2005; Kinloch et al., 2008; Jones et al., 2009).

Interestingly, *P. gingivalis* secrets an analogous enzyme of PAD, known as PPAD. At present, *P. gingivalis* is the only known human pathogen producing cysteine-dependent PPAD. PPAD contains N-terminal and C-terminal domains with different functions. N-terminal domains are involved in substrate binding, protein-protein, and protein-nucleic acid interactions. C-terminal domains function as the catalytic domain, which can convert arginine residue to citrullinate. *P. gingivalis* secretes PPAD in a secreted soluble state or outer membrane vesicle (OMW)-bound state. Thus, PPAD causes aberrant citrullination that contributes to inflammatory responses in PD and can be an etiological agent of RA.

Gingipains, primarily with arginine residues (RgpA and RgpB), are associated with PPAD because fewer citrullinated proteins are detected in the Rgp deletion mutant than the parent strains (Wegner et al., 2010b). In addition, there is increasing evidence that PPAD-catalyzed protein citrullination depends on the function of Rgp proteases. Rgp proteases can cleave polypeptides at arginine residues, freeing a C-terminal arginine residue for PPAD to citrullinate. It is worth noting that Rgps with arginine residues can be citrullinated by PPAD (Stobernack et al., 2016). In addition, PPAD can be autocitrullinated on arginine residue 352, reducing its activity and aid bacterial survival in periodontal pockets (Konig et al., 2015).

In addition to citrullinating bacterial proteins, PPAD has been shown to citrullinate specific human host proteins (Gabarrini et al., 2020). For example, PPAD can citrullinate the epidermal growth factor (EGF), blocking the recognition between the epithelium and the EGF signaling pathway molecules and impairing its ability to stimulate epidermal cell proliferation and migration (Pyrc et al., 2013). In addition, the pore-forming leukotoxin (LtxA) secreted by *A. actinomycetemcomitans* is involved in host protein citrullination by activating endogenous PAD2 and PAD4 in neutrophils (Engstrom et al., 2018). The mechanisms and pathways responsible for human protein citrullination might indicate associations between oral bacteria and several systematic diseases (atherosclerosis, rheumatic arthritis, and Alzheimer’s disease) (Olsen et al., 2018).

A recent analysis of *P. gingivalis* genomes of laboratory strains and clinical isolates unveiled a PPAD variant (PPAD-T2), with three amino-acid substitutions directly preceding the catalytic residue H^236^(G^231^N/E^232^T/N^235^D) compared with PPAD from the reference strain (PPAD-T1). Functionally, PPAD-T2 exhibits weaker substrate binding capability but higher catalytic rates than PPAD-T1. In addition, like PPAD-1, PPAD-T2 citrullinates arginine at the C-terminal domain of the targeted protein (Bereta et al., 2019).

### Other Post-translational Modifications

Additional PTMs have been identified and characterized in oral bacteria, with a vital role in bacterial physiological function and pathogenicity with the development of mass spectrometry. For example, a relatively recently discovered PTM is succinylation. Lysine succinylation has been defined as the transfer of a succinyl group to a lysine residue of the target protein, mediated either enzymatically or non-enzymatically with succinyl-CoA as the donor. Succinylation at lysine residues changes lysine’s charge from +1 to −1 and introduces a relatively larger structure moiety, leading to a mass increase of 100 Da, expected to contribute to significant changes in protein structure and function. Over the past few years, lysine succinylation has been identified in a few oral bacterial species. An example is *P. gingivalis*. Several important virulence factors, gingipains, fimbriae, RagB, and PorR, occur in lysine succinylation (Wu et al., 2019). Furthermore, protein succinylation and acetylation were extensively overlapped in *P. gingivalis*, including PGN_0377, PGN_0457, PGN_0497, PGN_0723, PGN_0724, PGN_0725, PGN_1176, PGN_1178, PGN_1341, and PGN_1367, which play a crucial role in the ribosome and metabolic processes (Zeng et al., 2020).

*S*-glutathionylation is the specific PTM of protein cysteine thiols by incorporating tripeptide glutathione (GSH), in which cysteine thiol and glutathione are classified as low-molecular-mass free (non-protein) thiols (Dalle-Donne et al., 2009). Thiols, organic sulfur derivatives (mercaptans), play a central role in coordinating the antioxidant signaling pathways. As a result, protein *S*-glutathionylation usually occurs in response to oxidative stress and prevents irreversible oxidation of cysteine residue, which is vital for maintaining intracellular redox homeostasis. A recent report showed that a thioredoxin-like protein (Tlp) is *S*-glutathionylated at Cys 41 residue, protecting *S. mutans* from reactive oxygen species (ROS) derived from hostile bacteria, e.g., *S. sanguinis* and *S. gordonii* (Li et al., 2020).

## Conclusion and Perspectives

As outlined in this review, PTMs are identified extensively in oral bacteria, with a profound influence on bacterial physiological processes and virulence regulation by altering the physicochemical nature of protein structure, activity, localization, and biomolecule interactions (Table 1). It is worth noting that protein modification machinery differs significantly in different bacterial species. For example, arginine phosphorylation is a protein degradation signal in the phylum *Firmicutes*, but so far, it has not been detected in other oral bacteria. Many oral bacterial PTMs depend on the metabolic status and specific products of metabolism. Acetylation is mainly catalyzed by acetyltransferase enzymatically and also by AcP non-enzymatically. However, more and more acetyl derivatives have been identified to participate in acetylation non-enzymatically in oral bacterial species, such as Ac-CoA and acetyladenylate, indicating that PTMs affect the intermediate products of primary metabolism, which reversibly regulate PTMs.

In general, a single protein can be modified by multiple PTMs either at the same or different sites. For example, lysine can be modified by a broad class of acylations, such as acetylation and succinylation. It has been demonstrated that both acetylation and succinylation modifications could occur in gingipains, fimbriae, RagB, and PorR, which are important virulence factors of *P. gingivalis*. The crosstalk between acetylation and phosphorylation was observed on RprY, and different modifications exert different biological functions. The acetylation of RprY reduces its ability to bind to the promoter of *nqrA*, while phosphorylation of RprY enhances its binding to the promoter of *nqrA*. Therefore, the crosstalk between different PTMs and their distinct and overlapping roles in regulating different physiological functions should be investigated further.

Many virulence- and survival-related proteins have been demonstrated to be modified in oral bacteria with recent advances in genomics and proteomics, and these studies suggest that PTMs play a critical role in bacterial virulence, survival, and pathogenicity. However, PTMs studies in oral bacterial species have mainly focused on a small number of species and strains, limiting the ability to explore the relationship between PTMs and bacterial functions and their regulatory mechanisms. For a complete understanding of the regulatory roles of PTMs, more studies will have to uncover the connection between the known modifications and their specific enzymes and vice versa. In conclusion, identification and characterization of PTMs not only contributes to uncover their roles and underlying regulatory mechanisms in oral bacterial virulence, adaption and resistance, but will open new avenues for the treatment of oral infectious diseases.

## Author Contributions

QM and QZ drafted and wrote the manuscript. JZ and YuL critically reviewed the manuscript and improved it. YC, SY, JH, YaL, and TG participated in manuscript correction. All authors gave final approval and agreed to be accountable for all aspects of the work.

## Conflict of Interest

The authors declare that the research was conducted in the absence of any commercial or financial relationships that could be construed as a potential conflict of interest.

## Publisher’s Note

All claims expressed in this article are solely those of the authors and do not necessarily represent those of their affiliated organizations, or those of the publisher, the editors and the reviewers. Any product that may be evaluated in this article, or claim that may be made by its manufacturer, is not guaranteed or endorsed by the publisher.
